# Dispersion and oviposition of *Aedes albopictus* in a Brazilian slum: Initial evidence of Asian tiger mosquito domiciliation in urban environments

**DOI:** 10.1371/journal.pone.0195014

**Published:** 2018-04-23

**Authors:** Tania Ayllón, Daniel Cardoso Portela Câmara, Fernanda Cristina Morone, Larissa da Silva Gonçalves, Fábio Saito Monteiro de Barros, Patrícia Brasil, Marilia Sá Carvalho, Nildimar Alves Honório

**Affiliations:** 1 Laboratório de Doenças Febris Agudas, Instituto Nacional de Infectologia Evandro Chagas/Fiocruz, Rio de Janeiro, Brasil; 2 Núcleo Operacional Sentinela de Mosquitos Vetores-Nosmove/Fiocruz, Rio de Janeiro, Brasil; 3 Laboratório de Mosquitos Transmissores de Hematozoários, Instituto Oswaldo Cruz, Rio de Janeiro, Brasil; 4 Departamento de Zoologia, Universidade Federal de Pernambuco, Recife-PE, Brasil; 5 Programa de Computação Científica PROCC/Fiocruz, Rio de Janeiro, Brasil; University of California, Davis, UNITED STATES

## Abstract

*Aedes albopictus*, originally considered as a secondary vector for arbovirus transmission, especially in areas where this species co-exist with *Aedes aegypti*, has been described in most regions of the world. Dispersion and domiciliation of *Ae*. *albopictus* in a complex of densely urbanized slums in Rio de Janeiro, Southeastern Brazil, was evidenced. In this study, we tested the hypotheses that 1) *Ae*. *albopictus* distribution in urban slums is negatively related to distance from vegetation, and 2) these vectors have taken on a domestic life style with a portion of the population feeding, ovipositing, and resting indoors. To do this, we developed an integrated surveillance proposal, aiming to detect the presence and abundance of *Aedes* mosquitoes. The study, based on a febrile syndrome surveillance system in a cohort of infants living in the slum complex, was performed on a weekly basis between February 2014 and April 2017. A total of 8,418 adult mosquitoes (3,052 *Ae*. *aegypti*, 44 *Ae*. *albopictus*, 16 *Ae*. *scapularis*, 4 *Ae*. *fluviatilis* and 5,302 *Culex quinquefasciatus*) were collected by direct aspiration and 46,047 *Aedes* spp. eggs were collected by oviposition traps. The Asian tiger mosquito, *Ae*. *albopictus*, was aspirated in its adult form (n = 44), and immature forms of this species (n = 12) were identified from the eggs collected by the ovitraps. In most collection sites, co-occurrence of *Ae*. *aegypti* and *Ae*. *albopictus* was observed. Key-sites, such as junkyards, thrift stores, factories, tire repair shops and garages, had the higher abundance of *Ae*. *albopictus*, followed by schools and households. We collected *Ae*. *albopictus* at up to 400 meters to the nearest vegetation cover. The log transformed (n+1) number of females *Ae*. *albopictus* captured at each collection point was inversely related to the distance to the nearest vegetation border. These results show that *Ae*. *albopictus*, a competent vector for important arboviruses and more commonly found in areas with higher vegetation coverage, is present and spread in neglected and densely urbanized areas, being collected at a long distance from the typical encounter areas for this species. Besides, as *Ae*. *albopictus* can easily move between sylvatic and urban environment, the entomological monitoring of *Ae*. *albopictus* should be an integral part of mosquito surveillance and control. Finally, key-sites, characterized by high human influx and presence of potential *Aedes* breeding sites, should be included in entomological monitoring.

## Introduction

Different arboviruses, such as dengue, chikungunya, Zika and yellow fever are transmitted to humans by mosquitoes of the genus *Aedes* (Meigen 1818), particularly *Ae*. *aegypti* (Linnaeus, 1762) and *Ae*. *albopictus* (Skuse, 1894), two invasive and frequently sympatric species. Although *Ae*. *albopictus* is considered to have a low capacity to transmit pathogens (as arboviruses) to humans, it has been demonstrated the potential role of this species in dengue, chikungunya and Zika virus transmission and outbreaks [1,2]. The domestic form of *Ae*. *aegypti* is highly anthropophilic, predominating in urban and suburban areas, where households and humans are abundant. However, *Ae*. *aegypti* is often found in transition areas between highly urbanized and urban forest, which might serve somehow as a refuge [3,4,5,6,7]. Furthermore, *Ae*. *albopictus* is typically more common in areas with higher vegetation coverage and more scattered human populations, but it has also been described in transitional environments with relatively low vegetation cover and frequently coexisting with *Ae*. *aegypti* [3,5,7,8,9]. Knowledge of the species habitat and environmental determinants is essential for predicting *Ae*. *aegypti* and *Ae*. *albopictus* presence and abundance in an area, which might impact arboviruses transmission. In this study, we tested the hypotheses that 1) *Ae*. *albopictus* distribution in urban slums is negatively related to distance from vegetation, and 2) *Ae*. *albopictus* has taken on a domestic life style with a portion of the population feeding, ovipositing, and resting indoors.

## Materials and methods

**Fig 1:**
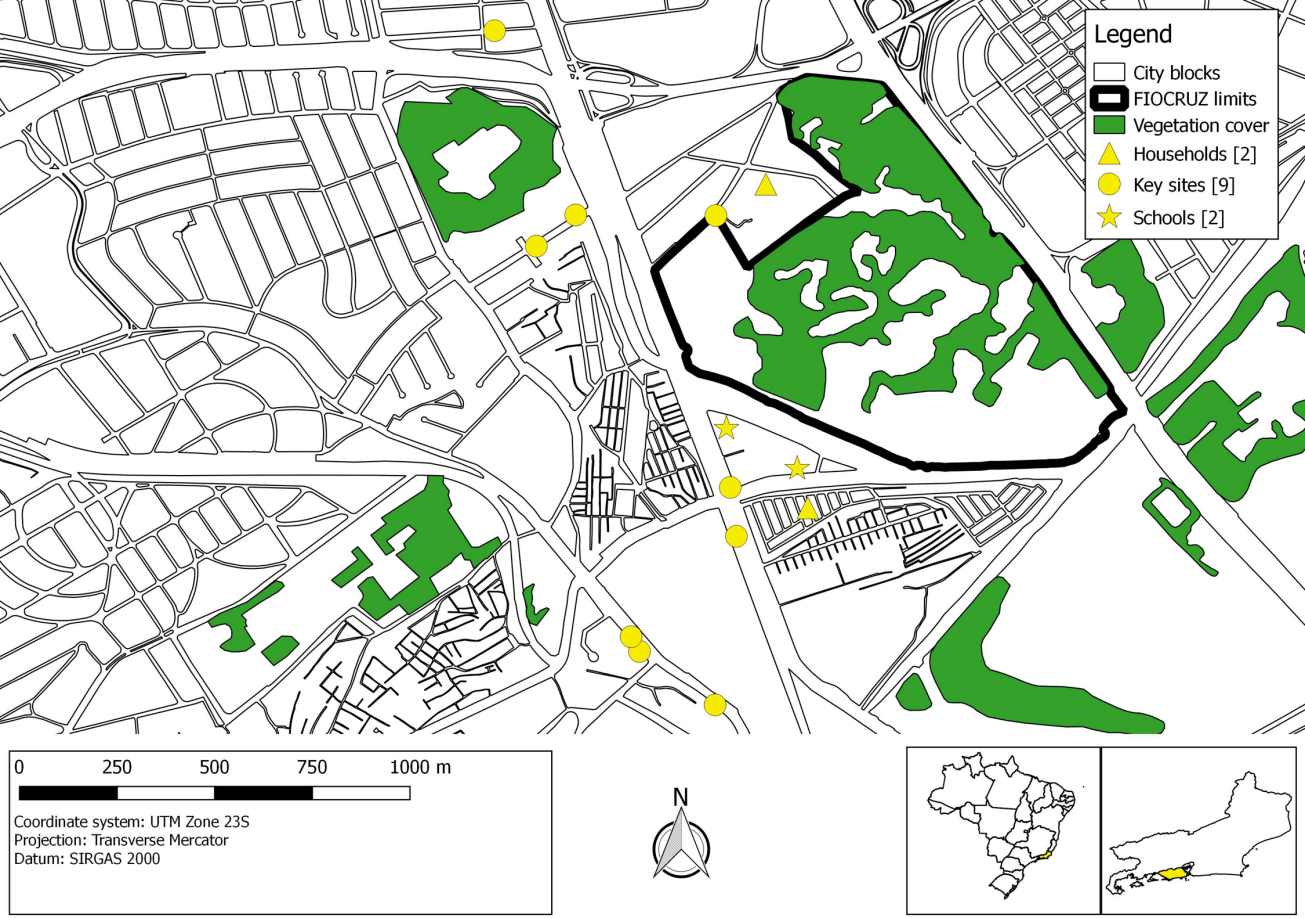
Spatial distribution of collections of *Aedes albopictus* adults in Manguinhos, Rio de Janeiro. Yellow triangles, circles, and stars represent the households, key-sites and schools, respectively, where *Ae*. *albopictus* adults were collected.

The study was conducted in Manguinhos (22° 52' 44,2 S 43° 14' 42,0 W), a low income urban slum complex comprised by 16 different densely urbanized communities. Within an area of 261 square kilometers, a population of 36,160 inhabitants (138 inhabitants per km^2^) live in 10,816 households [10], characterized by a crowded housing, narrow alleys, inadequate sanitation, irregular domestic water supplies and haphazard waste management. Violence and constant police incursions make Manguinhos a difficult neighborhood for research activities and entomological monitoring. Low vegetation is common in Manguinhos, although there are some green delimited areas in the community, such as the Fiocruz campus (Fig 1), a river, and other waterways.

A mosquito surveillance integrating a large-scale dengue infant cohort study [9] was conducted from February 2014 to April 2017 in the study area. Ethical clearance was obtained from the Ethical Committee in Research (CAAE: 13202113.1.0000.5240) from the National School of Public Health, Oswaldo Cruz Foundation, Ministry of Health, Brazil. Each participant signed informed consent. Adult mosquito collections were performed weekly integrated to the cohort study. We used portable backpack aspirators in several collection sites: households, schools and key-sites (such as junkyards, thrift stores, factories, tire repair shops and garages), the last two defined as non-residential properties suitable for the maintenance of vector infestation. Households were visited after the report of fever in any children followed-up in the cohort study. Schools and key-sites, selected in strategic areas in the vicinity of the fever cases, were characterized by high human influx and presence of potential *Aedes* breeding sites. Adult mosquito sampling was performed for 15–20 minutes in each collection site. All sampling was performed by the same technicians throughout the whole study period. Aspirations were positive when at least one mosquito was collected. Adult mosquitoes were counted, sexed and identified to species level using the taxonomic key of Consoli and Lourenço-de-Oliveira [11] and stored in freezer (-80°C). Oviposition was monitored placing 45 ovitraps weekly in the schools from October 2015 to May 2016, totaling 806 observations. Wooden paddles were collected weekly and inspected for the presence of eggs, which were counted and hatched to identify larvae species. Collection points were geo-referenced, and distance to the nearest vegetation cover was measured using QGIS 2.18. Shape files are publicly available and free to use at Rio de Janeiro’s Municipal Data Repository (http://www.armazemdedados.rio.rj.gov.br/). We modeled the log transformed (n+1) abundance of collected *Aedes* female mosquitoes and the distance from each collection point to the nearest vegetation patch in the study area using a simple linear model. Analyses were carried out in R and RStudio [12,13].

## Results

The mean daily temperature during the study period was 25.8°C (SD = 3.31°C, min = 14.3°C, max = 40.7°C). Rainfall was observed in 380 days (32.25% of the study period). The mean daily precipitation was 5.89mm (SD = 34.76mm, max = 514.60mm). Relative air humidity was 67.66% (SD = 8.99%, min = 41.85%, max = 91.58%). During the three-year period, a total of 244 households, 22 key-sites and nine schools were visited. During the study period, house index [14] was routinely evaluated by health department personnel and ranged from 0.19 to 2.16 in Manguinhos [15]. In the 1,214 visits performed, we identified 5,302 *Cx*. *quinquefasciatus* and 3,116 adult *Aedes* spp. among which 3,052 were *Ae*. *aegypti* (68% engorged), 44 were *Ae*. *albopictus* (58% engorged), 16 (0% engorged) were *Ae*. *scapularis* and 4 (0% engorged) were *Ae*. *fluviatilis* (Table 1). In the thirteen locations where *Ae*. *albopictus* was observed it co-occurred with *Ae*. *aegypti* in eleven locations (84.6%, Table 2). Thirty-eight *Ae*. *albopictus* adults (86.4%) were collected from seven of 22 key-sites, four (9.1%) from two of nine schools and two (4.5%) from two of 243 households surveyed (Table 2). Additionally, from 46,047 eggs collected from the nine schools (eclosion rate: 0.42%), 12 *Ae*. *albopictus* and 183 *Ae*. *aegypti* larvae were identified. The mean eggs/week per school varied between 13.2 and 233.8.

**Table 1:** Total number of mosquito adults collected in schools, key-sites and households in Manguinhos, Rio de Janeiro from February 2014 to April 2017.

Species	Collection sites		
	Schools	Key-sites	Households
***Ae*. *aegypti***			
Females	302	535	103
Engorged*	219	348	72
Males	400	1573	139
Subtotal	702	2108	242
***Ae*. *albopictus***			
Females	3	26	2
Engorged*	2	14	2
Males	1	12	0
Subtotal	4	38	2
***Ae*. *scapularis***			
Females	7	0	3
Engorged*	0	0	0
Males	5	1	0
Subtotal	12	1	3
***Ae*. *fluviatilis***			
Females	2	1	0
Engorged*	0	0	0
Males	0	1	0
Subtotal	2	2	0
***Cx*. *quinquefasciatus***			
Females	408	912	269
Males	1496	2106	111
Subtotal	1904	3018	380
**Total**	**2613**	**5178**	**627**

**Table 2:** Co-occurrence sites and number of *Aedes* spp. adults collected in the different sites positive for *Ae*. *albopictus* in Manguinhos complex during the study period (2014 to 2017).

Collection sites	Distance to the nearest green border (mts)	*Ae*. *albopictus*		*Ae*. *aegypti*		
		F	M	F	M
**Household #1**	269.3	1	-	-	-
**Household #2**	146.5	1	-	-	-
**Key-site #1**	183.6	-	1	1	5
**Key-site #2**	280.0	-	1	5	2
**Key-site #3**	167.1	10	-	19	62
**Key-site #4**	397.9	0	1	4	8
**Key-site #5**	259.0	1	1	1	1
**Key-site #6**	171.4	-	2	-	1
**Key-site #7**	101.7	4	1	3	66
**Key-site #8**	114.6	3	2	4	5
**Key-site #9**	223.0	8	3	12	53
**School #1**	165.9	1	1	5	14
**School #2**	137.8	2	-	-	4
**Total**		**31**	**13**	**54**	**221**

F, female; M, male; -, no specimens collected.

**Fig 2:**
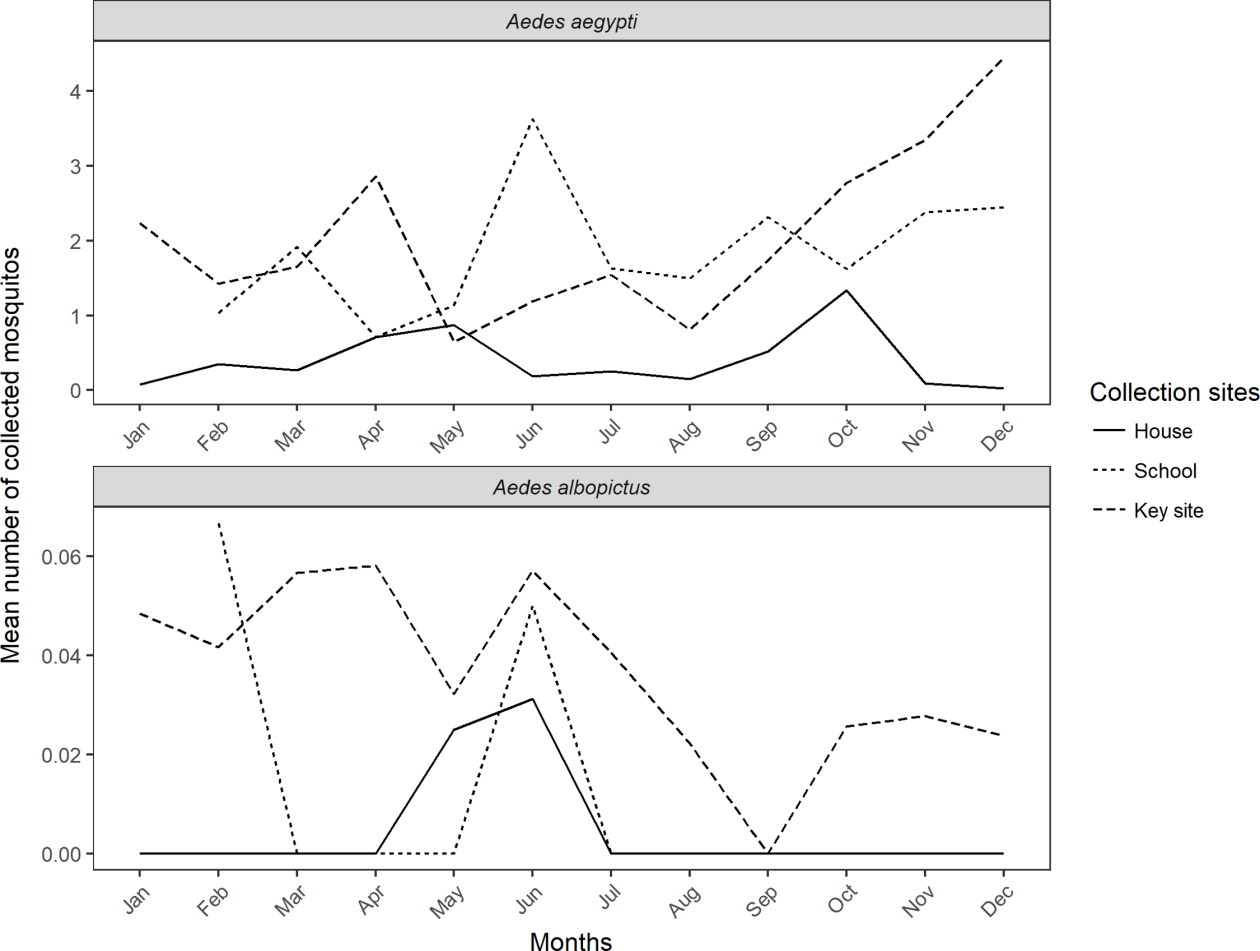
Monthly mean abundance of *Ae*. *aegypti* and *Ae*. *albopictus* during the three-year study period in Manguinhos, Rio de Janeiro. The figure shows the mean number of *Ae*. *aegypti* and *Ae*. *albopictus* mosquitoes collected per month during the study.

Both species were collected during all seasons. *Aedes aegypti* was more abundant during the wet season, peaking in December with abundance declining abruptly in May. *Aedes albopictus* peaked in June declining afterwards. For both species, more specimens were collected in the key-sites and schools. Throughout the study period *Ae*. *aegypti* was present in all three different collection sites, while *Ae*. *albopictus* maintained a positive mean only in key-sites. In schools and households, *Ae*. *albopictus* was less often found (Fig 2). Both *Ae*. *aegypti* and *Ae*. *albopictus* were strongly correlated (R = 0.87, p < 0.05).

**Fig 3:**
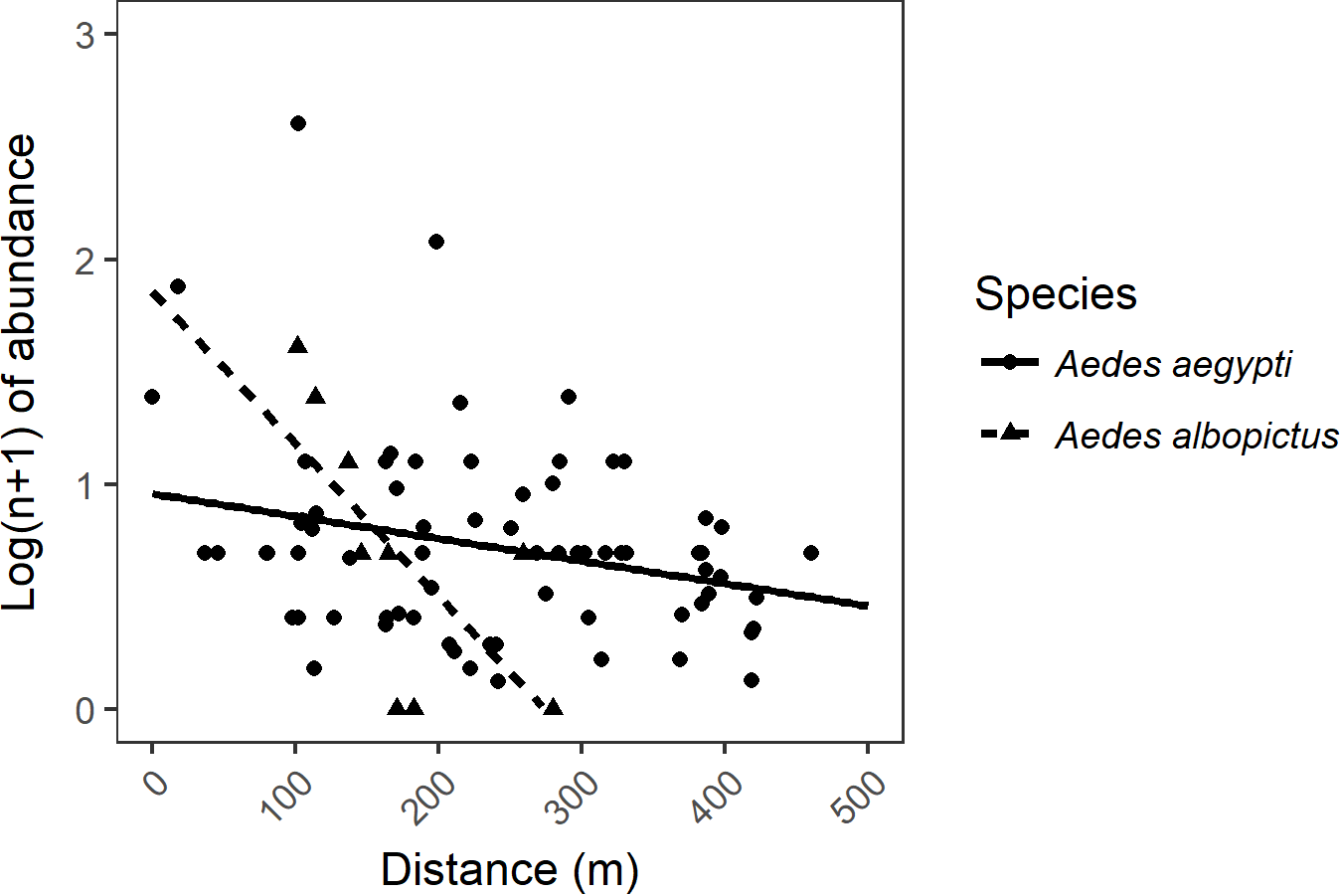
Model of the log-transformed (n+1) abundance of *Ae*. *aegypti* and *Ae*. *albopictus* females collected in the study area as related to the distance to the nearest vegetation patch, in meters. The figure shows the abundance of these species according to the distance, in meters, to the nearest vegetation border.

The number of *Ae*. *albopictus* adult females retrieved in the urbanized area fitted an exponential regression curve with the distance to the nearest vegetation border (R = 0.65; p < 0.0001). The log transformed (n+1) number of females *Ae*. *albopictus* captured at each collection point was inversely related to the distance to the nearest vegetation border (Fig 3). The regression curve parameters estimated were: y = 260.5629*exp (-0.2545*x), where y represents the distance to the nearest vegetation border and x the number of female *Ae*. *albopictus* retrieved. Regression curve parameters estimated for the linear regression curve: y = a+bx, were a = 253.3969 and b = -43.4897. The number of *Ae*. *aegypti* adult females retrieved near the vegetated areas also fitted an exponential regression curve, with the number of females decreasing exponentially with the distance to the nearest vegetation border (p < 0.0001). The number of females of *Ae*. *aegypti* captured at each collection point weighted according to the number of collections performed at each site, was inversely related to the distance to the nearest vegetation border (Fig 3). The regression curve parameters estimated were: y = 307.6928*exp (-0.2368*x), where y represents the distance to the nearest vegetation border and x the number of female *Ae*. *aegypti* retrieved (Fig 3).

## Discussion

In our study, the entomological survey followed reports of febrile children, in a routine entomological surveillance. Both *Ae*. *aegypti* and *Ae*. *albopictus* were collected indoors in an urban endemic area for dengue, Zika and chikungunya, with the number of females of similar magnitude (Table 2). Although *Ae*. *albopictus* is typically not commonly found in densely urbanized slums [5], we identified 44 adults and 12 immature forms of this species during the three-year survey in Manguinhos. *Aedes albopictus* has adapted well to suburban and urban environments, and has been described as the sole vector in urban areas in China and Italy [16,17]. Moreover, there is evidence of a geographical variation in the behavior of this species, with gravid females captured indoors in Italy [18]. The collection of *Ae*. *albopictus* adults in densely urbanized slums as Manguinhos complex seems to emphasize the dispersion (a statistical term that describes the distribution of organisms over a landscape) [19] of this species, and could be indicative of an increased establishment of this species in anthropogenic-influenced environments, as occurred for *Ae*. *aegypti* [1,20]. In addition, our results support the evidence of an initial domiciliation by *Ae*. *albopictus*, defined as the process by which a species occupy niches in the anthropic environment (feeding, resting, and perhaps mating indoors) [21].

Most of the *Ae*. *albopictus* adults were collected from key-sites, typically described as highly favorable to *Ae*. *aegypti* infestation as shown by the positive and strong correlation between both species [22]. The surveillance and monitoring of such areas are essential to inform vector control strategies [22,23,24]. The fragile infrastructure of key-sites favors *Ae*. *aegypti* proliferation [8], but has not yet been linked to *Ae*. *albopictus* production. In fact, Manguinhos complex promotes high vector infestation levels through poor sanitation, interrupted water supply and high human population density. In locations where *Ae*. *albopictus* was collected (5.1% of sites surveyed), *Ae*. *aegypti* was also present in 84.6% (11 of 13) of the locations, showing a clear pattern of co-occurrence, i.e. *Ae*. *aegypti* was present in most of the sites where *Ae*. *albopictus* was collected. In a previous entomological survey in the same area, both species co-occurred at the transition zone between the forest and the densely populated region [5].

In another entomological survey, low numbers of immature *Ae*. *albopictus* were found in Favela do Amorim, one of the 16 slums that composes Manguinhos complex and which also surrounds the forested area in the Fiocruz campus [25]. In our study, the presence of *Ae*. *albopictus* adult females and males, together with eggs and larvae, led us to conclude that this species may be establishing itself in the slums of Manguinhos.

The finding of *Ae*. *albopictus* inside the households, where febrile cases were reported, clearly indicates that this species has a tendency toward domesticity that may not be as strong as that of *Ae*. *aegypti*, but that nevertheless could be of epidemiological importance. Indoor residence of this species highlights the need of maintaining entomological and epidemiological surveillance in vulnerable areas. This is of utmost importance, since we demonstrated the presence of Zika virus in engorged *Ae*. *aegypti* mosquitoes in a key-site where *Ae*. *albopictus* was found, in the same densely urbanized slum, before the first case of autochthonous Zika virus disease was diagnosed in Rio de Janeiro city [24].

Since both *Ae*. *aegypti* and *Ae albopictus* share the same larval habitats, it has been suggested that their coexistence may be a transient phenomenon, that should be followed by the reduction or displacement of one of the two species through interspecific competition during larval stages [1,26,27] or through asymmetric reproductive interference via interspecific mating. This last circumstance is also known as satyrization, that is a form of reproductive interference where males of *Ae*. *albopictus* mate with females of *Ae*. *aegypti* resulting in no offspring and permanent sterilization of the cross-mated females [28,29]. However, our monitoring of the current study areas during the last 15 years suggests that the two species may have reached a relative steady state of coexistence in urban areas of Manguinhos, Rio de Janeiro. In addition, we recently showed the lack of major competitive displacement of Brazilian *Ae*. *albopictus* males (including Manguinhos strain) to satirize *Ae*. *aegypti* females, suggesting that the low satyrization potential of Brazilian *Ae*. *albopictus* males may account for the lack of displacements of *Ae*. *aegypti* [29]. A previous study showed that this coexistence shows large seasonal fluctuations in both pupal productivity and interspecific competition in the study area, favoring *Ae*. *albopictus* over *Ae*. *aegypti*. Even though the study shows a clear advantage for *Ae*. *albopictus*, seasonal fluctuation of the interspecific competition effects over *Ae*. *aegypti* are not sufficient to displace this species in the study area [7]. It has been shown that *A*. *albopictus* is superior to *Ae*. *aegypti* in resource competition, maintaining greater population growth at higher combined densities [7,30,31], as well as producing greater survivorship during periods of low food availability [32].

In the present paper, we have found a similar pattern for spatial distribution of *Ae*. *albopictus* females within the urban area, with mosquitoes collected at almost 400 meters to the nearest vegetation area. Previous studies showed that gravid *Ae*. *albopictus* are capable of dispersing at least 800 m in urban areas [33], and that their larvae showed competitive advantages over *Ae*. *aegypti* [34,7]. Our results suggest that *Ae*. *albopictus*, a competent vector for important arboviruses, including dengue (DENV), chikungunya (CHIKV), Zika (ZIKV) and yellow fever (YFV) [35,36,37], may spread into neglected and densely urbanized areas, if close to vegetated areas. In addition, as this species tends to shelter outside houses [4], are capable of dispersing great distances inside forests near human dwellings and can easily move between sylvatic and urban environments [38], there is an urgent need to establish entomological surveillance protocols targeting this species.

The results obtained in this study show the global importance of maintaining entomological monitoring of *Ae*. *albopictus* as a part of surveillance and control programs. This is especially true in Brazil and elsewhere in the Americas where *Ae*. *albopictus* might participate in the spillback of arboviruses to enzootic cycles much in the same way as happened to YFV in the last few centuries [1,20,39]. In fact, preliminary evidence shows that ZIKV might be already circulating among neotropical nonhuman primates in Brazil [40]. Besides, this arbovirus has already been detected in wild-caught *Ae*. *albopictus* from Bahia, Brazil [41]. Thus, entomological surveillance studies integrate with host-seeking behavior of *Ae*. *albopictus* should be investigated inside densely urbanized slums in order to determine whether the presence of *Ae*. *albopictus* in slums near vegetated border has an epidemiological importance in the transmission dynamics of these arboviruses.

## Conclusions

Densely urbanized slums favor the permanent circulation of mosquitoes, humans and viruses. Continuous longitudinal monitoring is essential in these vulnerable areas in spite of all challenges such as limited access, violence and floodings. Moreover, key-sites, with high human concentration, mobility, and presence of potential *Aedes* breeding sites, should be included in entomological monitoring. Concomitantly, due to the increasing evidence confirming *Ae*. *albopictus* as an efficient viral vector, it would be necessary to extend the entomological monitoring for *Ae*. *albopictus* mosquito species. This species has been shown to be a primary vector for arboviruses in different countries and has progressively established in urban areas. However, it has not been a target of surveillance programs in Brazil yet. Finally, this study points out the great importance of integrated studies, since they reinforce the virological, entomological and epidemiological approaches.

## Supporting information

S1 Table Excel file containing the original data used for Tables 1 and 2. The table contains the number of *Aedes* aegypti and *Ae*. *albopictus* mosquitoes collected during the study period and the distances, in meters, to the nearest green border.
(XLSX)
